# TGF-β induced EMT and stemness characteristics are associated with epigenetic regulation in lung cancer

**DOI:** 10.1038/s41598-020-67325-7

**Published:** 2020-06-30

**Authors:** Bit Na Kim, Dong Hyuck Ahn, Nahyeon Kang, Chang Dong Yeo, Young Kyoon Kim, Kyo Young Lee, Tae-Jung Kim, Sug Hyung Lee, Mi Sun Park, Hyeon Woo Yim, Jong Y. Park, Chan Kwon Park, Seung Joon Kim

**Affiliations:** 1 Department of Internal Medicine, College of Medicine, The Catholic University of Korea, Seoul, Korea; 2 Postech-Catholic Biomedical Engineering Institute, St. Mary’s Hospital, College of Medicine, The Catholic University of Korea, 222, Banpo-daero, Seocho-gu, Seoul, 06591 Korea; 3 Department of Hospital Pathology, College of Medicine, The Catholic University of Korea, Seoul, Korea; 4 Department of Pathology, College of Medicine, The Catholic University of Korea, Seoul, Korea; 5 Department of Biostatistics, Clinical Research Coordinating Center, The Catholic University of Korea, Seoul, Korea; 6 Department of Cancer Epidemiology, Moffitt Cancer Center, Tampa, FL USA

**Keywords:** Cancer, Cell biology

## Abstract

Transforming growth factor-β (TGF-β) promotes tumor invasion and metastasis by inducing epithelial-mesenchymal transition (EMT). EMT is often related with acquisition of stemness characteristics. The objective of this study was to determine whether EMT and stemness characteristics induced by TGF-β might be associated with epigenetic regulation in lung cancer. A human normal lung epithelial cell line and four lung cancer cell lines were treated with TGF-β. Transcriptome analysis of BEAS-2B and A549 cells incubated with TGF-β were analyzed through next-generation sequencing (NGS). Western blotting was carried out to investigate expression levels of epithelial and mesenchymal markers. Wound healing and Matrigel invasion assay, sphere formation assay, and in vivo mice tumor model were performed to evaluate functional characteristics of EMT and stemness acquisition. To investigate whether activation of EMT and stem cell markers might be involved in epigenetic regulation of lung cancer, experiment using a DNA methyltransferase inhibitor (5-azacytidine, AZA), methylation-specific PCR (MSP) and bisulfite sequencing were performed. NGS revealed changes in expression levels of EMT markers (E-cadherin, N-cadherin, fibronectin, vimentin, slug and snail) and stem cell markers (CD44 and CD87) in both BEAS-2B and A549 cells. Functional analysis revealed increased migration, invasion, sphere formation, and tumor development in mice after TGF-β treatment. Expression of slug and CD87 genes was activated following treatment with AZA and TGF-β. MSP and bisulfite sequencing indicated DNA demethylation of slug and CD87 genes. These results suggest that TGF-β induced EMT and cancer stemness acquisition could be associated with activation of slug and CD87 gene by their promoter demethylation.

## Introduction

Although improvements have been made in cancer treatment, lung cancer remains the leading cause of cancer death worldwide. The poor prognosis is due to its diagnosis at advanced stage of the disease^1,2^. Failure in treatment is related with cancer recurrence and metastasis. It has been reported that both epithelial–mesenchymal transition (EMT) and acquisition of cancer stemness play important roles in the invasion, metastasis, and chemoresistance of solid tumors^3,4^.

Transforming growth factor-beta (TGF-β) regulates invasion and metastasis through loss of epithelial markers and gain of mesenchymal markers. TGF-β induced EMT is a major feature of EMT invasiveness and metastasis for tumor progression^5,6^. During the transition, epithelial cells can obtain invasive and migratory properties to become cells having stemness characteristics^3^.

Cancer stem cell (CSC) has the ability to self-renew and initiate tumor formation. Thus, it is intrinsically resistant to therapy. EMT inducers such as TGF-β, Wnt, Notch, NF-kB, and ERK/MAPK pathways can promote stem cell characteristics of solid tumors^7–10^. During EMT and stemness acquisition, epigenetic mechanisms such as DNA methylation and histone modifications are involved in the regulation of EMT and stemness-related genes^11–15^. However, whether EMT and stemness characteristics induced by TGF-β might be associated with epigenetic regulation in lung cancer remains unclear. Thus, the aim of the present study was to evaluate the effect of TGF-β induced EMT on stemness acquisition of lung cancer cells and determine the possible epigenetic mechanisms involved in the development of lung cancer.

## Methods

### Cell culture and transcriptome analysis

A human normal lung epithelial cell line and four lung cancer cell lines were purchased from the American Type Culture Collection (ATCC, Manassas, VA, USA). BEAS-2B cells were cultured in DMEM/F/12 medium. A549, H292, H226, and H460 cells were maintained in RPMI 1640 medium. Each of the medium was supplemented with 10% fetal bovine serum (FBS), 100 U/mL penicillin, 100 μg/mL streptomycin, and 250 ng/mL amphotericin B. Cells were maintained at 37 ℃ in a 5% CO2 humidified atmosphere. To analyze the effect of TGF-β, cells were treated with 10 ng/mL TGF-β (R&D Systems, Minneapolis, MN, USA).

We used not only representative NSCLC cell lines (one adenocarcinoma cell line, NCI-A549; one squamous cell carcinoma cell line, NCI-H226), but also other lung cancer cell lines (one large cell carcinoma cell line, NCI-H460; one mucoepidermoid pulmonary carcinoma cell line, NCI-H292) for the supportive information. We want to confirm the effect of TGF-β on EMT and stemness acquisition, which is a general feature regardless of aggressiveness of lung cancer cell lines including normal lung cell line although BEAS-2B might not the best control for adenocarcinomas.

**Figure 1:**
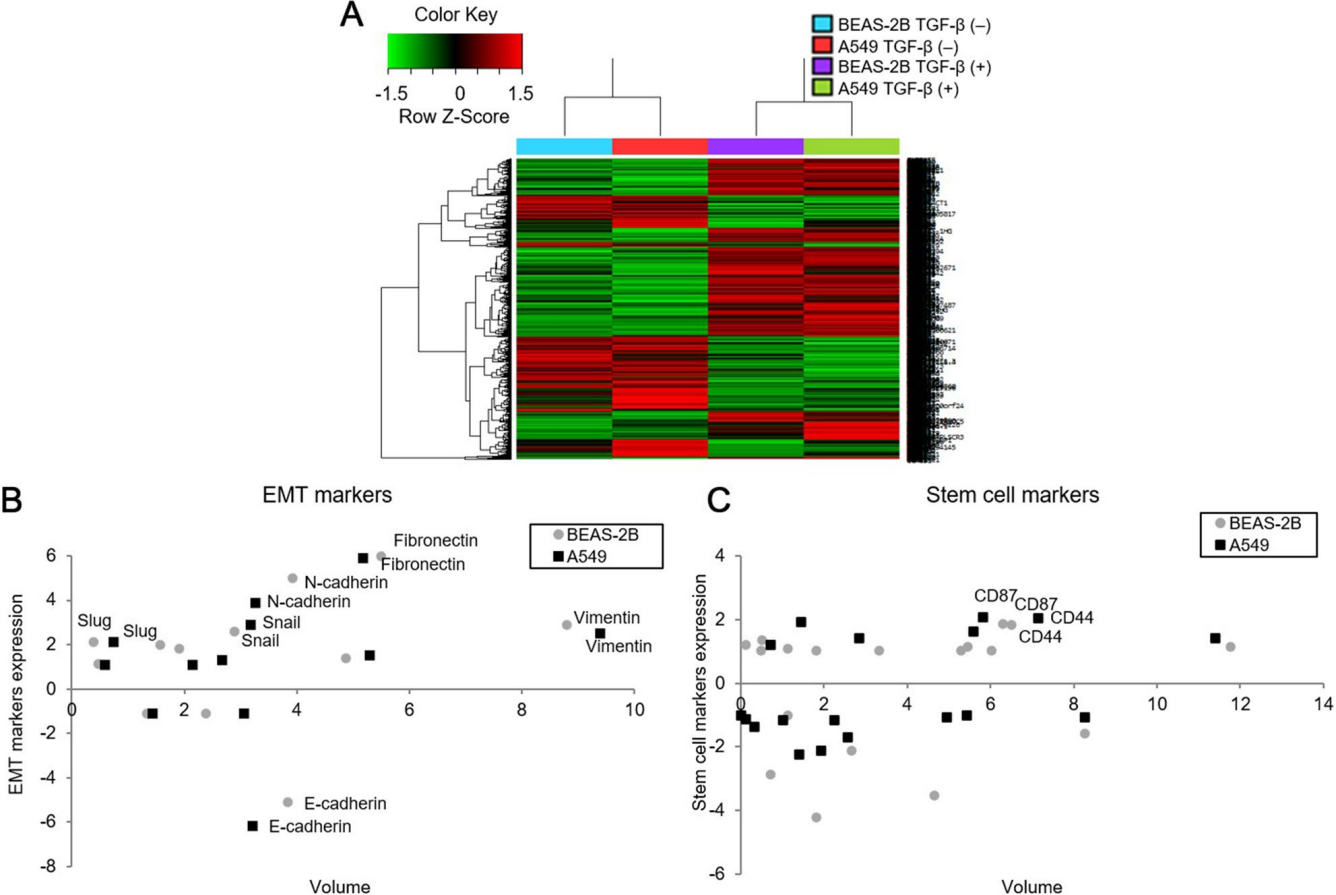
Transcriptome analysis using next-generation sequencing in BEAS-2B and A549 cells treated with TGF-β for 72 h to screen EMT and stemness genes. (**A**) A distinct separation of mRNA expression patterns of cells treated with or without TGF-β was indicated by heat map of hierarchical clustering. (**B**) mRNA expression levels of N-cadherin, fibronectin, Vimentin, slug, and snail were increased in lung cells treated with TGF-β whereas those of E-cadherin were decreased in cells treated with TGF-β. (**C**) Expression levels of stem cell markers (CD44 and CD87) were enhanced following TGF-β treatment.

Transcriptomes of BEAS-2B and A549 cells treated with TGF-β for 72 h were analyzed as described previously^16^. Transcriptome analysis by next-generation sequencing (NGS) in Fig. 1 was based on two samples (BEAS-2B and A549) to screen EMT and stemness genes induced by TGF-β treatment.

### Western blot analysis

Cells were lysed using radioimmunoprecipitation assay (RIPA) buffer supplemented with protease inhibitors for 20 min. Proteins (20 μg) extracted from cells were separated on SDS-PAGE gel and transferred to a nitrocellulose membrane. The membrane was blocked for 1 h with blocking buffer (5% non-fat milk in PBS containing 0.1% Tween 20) and then incubated with primary antibodies dissolved in blocking buffer at 4 ℃ overnight. Membranes were washed with TBS-T buffer (PBS with 0.1% Tween 20) for 5 min three times and incubated with horseradish peroxidase (HRP)-conjugated secondary antibodies at RT for 1 h. The membrane was developed using an ECL Kit (Amersham Pharmacia Biotech, Little Chalfont, Buckinghamshire, UK) with X-ray film.

The primary antibodies used in this western blotting were as follows: E-cadherin (H-108, 1:1000, sc-7870; Santa Cruz Biotechnology, Inc.), N-cadherin (H-63, 1:1000, sc-7939; Santa Cruz Biotechnology, Inc.), fibronectin (EP5, 1:1000, sc-8422; Santa Cruz Biotechnology, Inc.), vimentin (V9, 1:1000, sc-6260; Santa Cruz Biotechnology, Inc.), slug (1:1000, GTX31749; GeneTex), snail (1:10000, GTX125918; GeneTex), and GAPDH (D4C6R, 1:1000, 97,166; Cell Signaling Technology).

### Migration, Matrigel invasion and sphere formation assay

Migration, Matrigel invasion and sphere formation assay were performed as described previously^16^. After overnight incubation of lung cells, wells were manually scratched using a 200-μL pipette tip on the bottom of the well. Migrated cells were cultured in serum-free medium with or without 10 ng/mL TGF-β for 72 h.

After treatment with TGF-β for 72 h, 2.5 × 10^4^ cells were seeded onto Matrigel-coated membrane with Falcon Cell Culture Inserts in 24-well plates (pore size, 8 μm; Corning Inc., Corning, NY, USA) containing serum-free medium. Inserts were placed into 24-well culture plates that contained medium with 5% FBS. The ImageJ software was used to count the number of invaded cells.

Lung cells were seeded at densities of 5 × 10^3^ cells per well in serum-free DMEM/F-12 medium supplemented with 1 × B27 (Gibco, Grand Island, NY), 20 ng/mL epidermal growth factor (EGF; PeproTech Inc., Rocky Hill, NJ), and 20 ng/mL fibroblast growth factor (FGF; PeproTech Inc., Rocky Hill, NJ) in Corning Ultra-Low Attachment 6 well plates (Corning Inc., Corning, NY). After treatment with TGF-β for 72 h, spheres were captured with an EVOS XL Core microscope (Advanced Microscopy Group, Bothell, WA, USA).

### Animal model

Mice experiments were carried out according to guidelines approved by the Institutional Animal Care and Use Committee of the Catholic University Medical School (Approval no. CUMC-2015-0080-01). Based on methods described in previous studies^17,18^, we prepared an animal subcutaneous implantation model using BALB/c nude mice for the analysis of stemness characteristics. After incubating BEAS-2B, A549, and H226 cells with TGF-β for 72 h, 1 × 10^5^, 1 × 10^6^, or 5 × 10^6^ cells were injected into each side of the mouse flank. Cells untreated with TGF-β were implanted on the left and cells treated with TGF-β were implanted on the right. Tumor development and growth were checked three times per week. At 2 weeks after injection, mice were sacrificed to obtain tumor samples.

### Treatment with a DNA methyltransferase inhibitor

Cells were incubated at a low density (1.0 × 10^5^ cells/well in 6-well plates) for 24 h before treatment. They were then treated with 5 µM 5-azacytidine (AZA, DNA methyltransferase inhibitor; Sigma, St. Louis, MO, USA) for 24 h. DMSO was used as a negative control.

### Real-time RT-PCR to analyze CD87 and slug expression

1 μg of RNAs extracted from BEAS-2B and A549 cells for each test were used to synthesize cDNAs. Expression levels of slug and CD87 were then quantified using GoTaq® qPCR Master Mix (Promega, Madison, WI, USA) and primers specific for slug and CD87 on an Exicycler™ 96 (Bioneer, Daejeon, Korea). PCR conditions were: an initial denaturation step of 95 °C for 5 min, followed by 40 cycles for 30 s at 95 °C, 60 °C, and 72 °C. Expression levels of slug and CD87 were normalized to that of GAPDH.

### Analysis of slug and CD87 promoter regions and sodium bisulfite conversion

For methylation-specific PCR and bisulfite sequencing, slug and CD87 primers were designed using Methyl Primer Express® Software Version 1.0 (Applied Biosystems, Foster City, CA, USA) and MethPrimer (www.urogene.org/methprimer) based on the location and structures of slug and CD87 CpG islands. DNA extracted from cells was treated with sodium bisulfite (EpiTect Bisulfite Kit; Qiagen, Seoul, Korea).

### Methylation-specific PCR

Methylation-specific PCR was performed to investigate slug and CD87 methylation in bisulfite-modified DNA. The following primer pairs were used to determine unmethylation levels of slug and CD87 genes (slug: forward, GGAAGCCCTGAGTAGCGCAGC; reverse, AGACAAAGGCGCCTGTGAGCG and CD87: forward, CACCAGCCGGCCGCGCCCC; reverse, CTCCCAGACGTTTTGCGAA). Methylation levels of slug and CD87 genes were analyzed with the following primer pairs (slug: forward, GGAAGTTTTGAGTAGCGTAGC; reverse, AAACAAAAACGCCTATAAACG and CD87: forward, TATTAGTCGGTCGCGTTTC; reverse, CTCCCAAACGTTTTACGAA). Both unmethylation and methylation levels were normalized against β-actin gene. Ratio of methylation level of target gene (slug or CD87) to that of β-actin was used to represent the relative level of unmethylation and methylation of promoter DNA (slug and CD87/β-actin). Bisulfite-modified DNA was amplified using GoTaq® Green Master Mix (Promega, Madison, WI, USA), 10 μM of each primer, and 250 ng of template DNA using MyCycler™ PCR machine (Bio-Rad, Hercules, CA, USA). PCR products were separated with 2% agarose gel and detected using ChemiDoc™ Imaging Systems (Bio-Rad, Hercules, CA, USA) and ImageJ software.

### Bisulfite sequencing PCR

Bisulfite sequencing PCR was evaluated using bisulfite-modified DNA at CD87 promoter region with the following primers: forward, TTTGGGGATAGAGTTGTGATT; reverse, CCTCAATTAAACCCTATTCCAA. PCR products were then sequenced.

### Statistical analysis

Data are presented as mean values ± standard error. Considering the small sample size, analyses were performed using non-parametric Wilcoxon rank-sum tests to identify significant differences between two groups. All reported P values are one-tailed P-value for a pre-specified direction in the alternative because the hypothesis of interest was only one-directional based on previous studies^19,20^ regarding the induction of EMT and stemness acquisition by TGF-β treatment. Statistical analyses were performed using SAS version 9.4 (SAS Institute, Cary, NC, USA).

## Results

### Transcriptome analysis following TGF-β treatment using NGS

To investigate the effect of TGF-β on mRNA expression in lung cells (BEAS-2B and A549), next-generation sequencing was performed for transcriptome analysis. Obvious differences in mRNA expression patterns were shown between cells treated with and without TGF-β (Fig. 1A). To analyze the effect of TGF-β on EMT and stemness characteristics, various EMT and stem cell markers were examined. Expression levels of E-cadherin, the epithelial marker, were decreased 5.1- to 6.1-fold while those of mesenchymal markers (N-cadherin, fibronectin, vimentin, slug and snail) were increased more than twofold in cells treated with TGF-β (Fig. 1B). Among stem cell marker candidates, CD44 and CD87 expression levels were increased around twofold following TGF-β treatment (Fig. 1C). Fold changes of various EMT and stem cell markers are presented in Table 1.

**Table 1:** Next-generation sequencing analysis for fold change and gene volume of EMT and stem cell marker expressions induced by TGF-β.

Gene	Fold change		Gene volume	
	BEAS-2B	A549	BEAS-2B	A549
EMT related				
E-cadherin	− 5.131572	− 6.16003	3.836665	3.210531
N-cadherin	4.99275	3.846411	3.915075	3.275512
Fibronectin	5.995764	5.927276	5.498908	5.162317
Vimentin	2.87307	2.532724	8.781097	9.386723
α-SMA	1.968616	1.050212	1.570309	2.143424
Slug	2.130321	2.102772	0.381627	0.742718
Snail	2.603393	2.879873	2.868386	3.170173
Twist1	− 1.095069	− 1.1355	1.340565	1.423161
Twist2	− 1.103786	− 1.14108	2.368498	3.055397
ZEB1	1.867336	1.335399	1.888146	2.674815
ZEB2	1.128909	1.072538	0.460226	0.588725
ZO-1	1.369319	1.524285	4.877554	5.275308
Stem cell related				
CD44	1.846255	1.998812	6.509078	7.17178
CXCR4	1.051758	− 1.13452	0.476323	0.121663
ABCG2	− 4.241183	− 2.25365	1.812526	1.415971
ALDH1A1	1.132919	1.393279	11.74794	11.41651
EpCAM	− 3.548091	− 2.12584	4.650325	1.94072
CD90	1.379704	1.9047	0.522715	1.466032
Nanog	1.184785	− 1.01217	0.112677	0.009034
SOX2	− 2.840429	− 1.42603	0.703701	0.328854
SSEA4	1.039819	− 1.2167	1.810111	2.276493
CD166	1.06801	1.618312	5.318266	5.592176
BMI-1	1.025095	1.377714	3.322621	2.855195
Nestin	− 1.01858	− 1.21427	1.13823	1.034249
Musashi-1	1.122545	1.180573	1.117478	0.757083
CD87	1.906373	2.066904	6.315332	5.832158
MET	1.155958	− 1.08475	5.463949	4.968498
SLC3A2	− 1.563981	− 1.08387	8.266959	8.284722
c-Myc	1.057811	− 1.04973	6.020233	5.450993
KLF4	− 2.110727	− 1.71194	2.649128	2.578687

### Expression of TGF-β induced EMT markers

**Figure 2:**
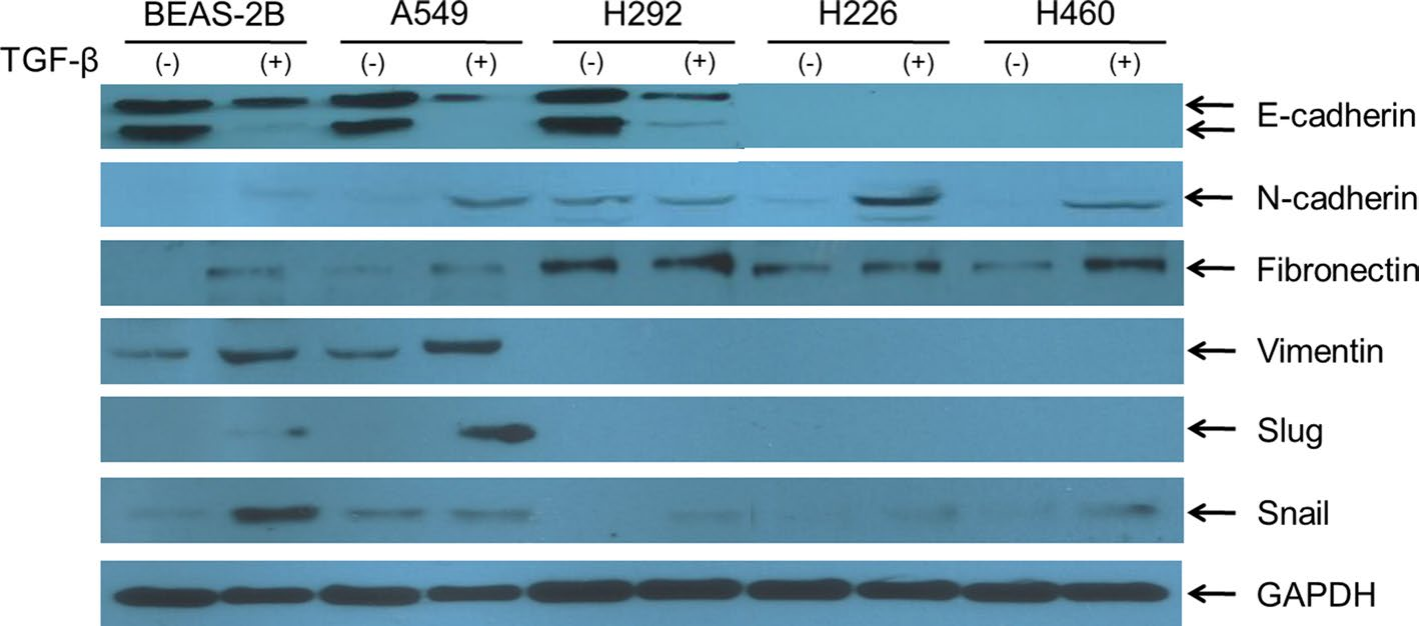
Expression of TGF-β induced EMT markers. E-cadherin expression levels were decreased in lung cells (BEAS-2B, A549 and H292) following TGF-β treatment whereas those of N-cadherin, fibronectin, vimentin, slug, and snail were increased, although their protein levels differed according to lung cells. Cropped images are displayed, uncropped blots are displayed in Supplementary Fig. S1.

The protein expression levels of each marker were evaluated with a series of cell lines, BEAS-2B, A549, H292, H226 and H460. Consistent with transcriptome analysis, E-cadherin expression was decreased in BEAS-2B, A549, and H292 cells treated with TGF-β. Expression levels of N-cadherin, fibronectin and snail were increased in BEAS-2B, A549, H292 and H460 cells and those of vimentin and slug were increased in BEAS-2B and A549 cells treated with TGF-β although their expression levels differed among lung cell lines (Fig. 2).

### Functional assessment of EMT following TGF-β treatment

**Figure 3:**
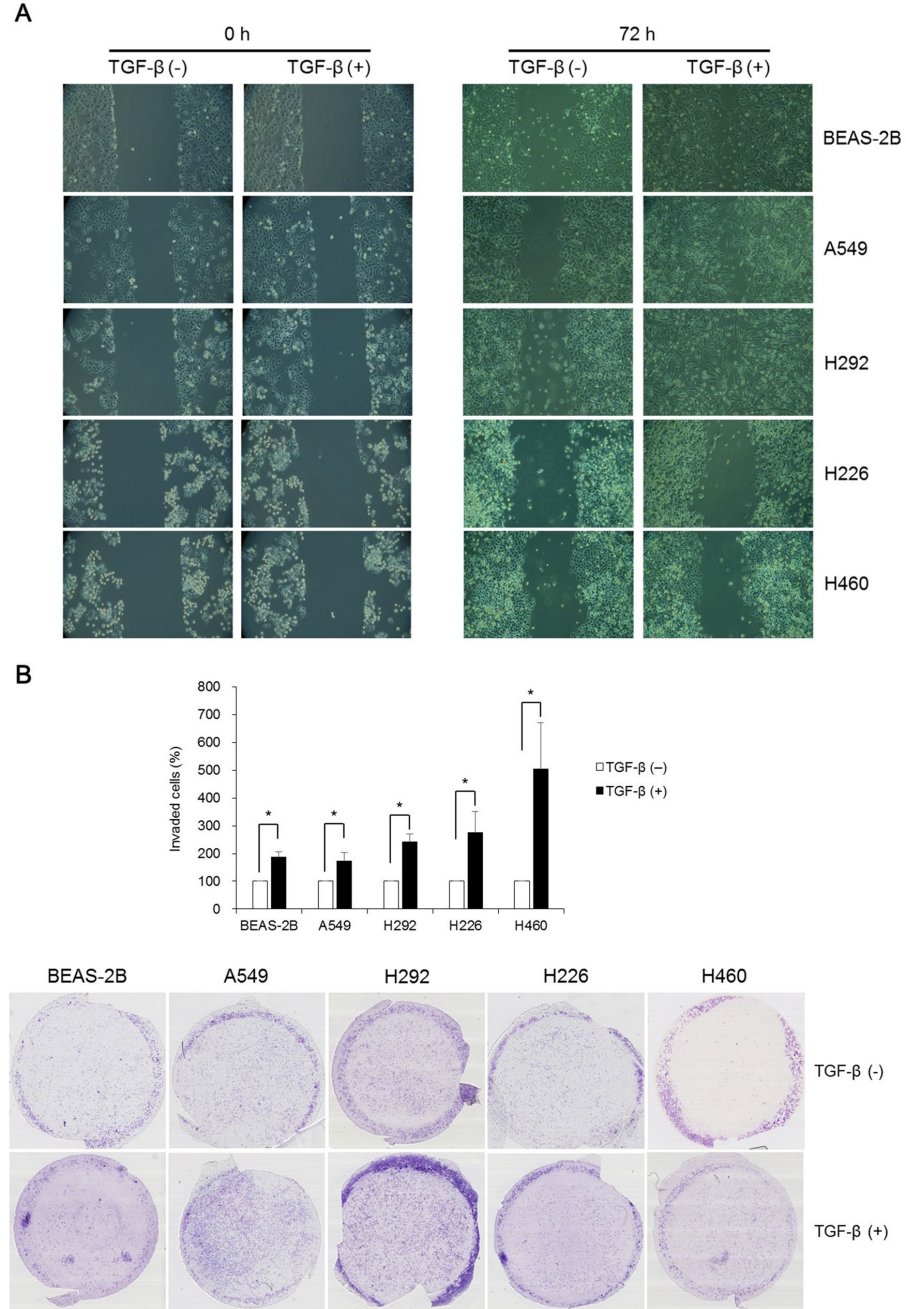
Functional analysis of EMT using wound healing and Matrigel invasion assays following TGF-β treatment. (**A**) Although the growth rate of lung cells treated with TGF-β was different, cell migration toward the center of scratched area was higher in lung cells induced by TGF-β. (**B**) Transwell invasion in BEAS-2B, A549, H292, H226, and H460 cells following TGF-β treatment was enhanced. Data are presented as mean ± SE (n = 4, *P < 0.05).

To examine whether TGF-β could augment migration and invasion, wound healing and Matrigel invasion assay were performed. Wound healing assay revealed that TGF-β increased migration of lung cells toward the center of the scratched area (Fig. 3A). Matrigel invasion assay indicated that TGF-β significantly increased transwell invasion of lung cells (Fig. 3B).

### Acquisition of stemness in vitro and in vivo

**Figure 4:**
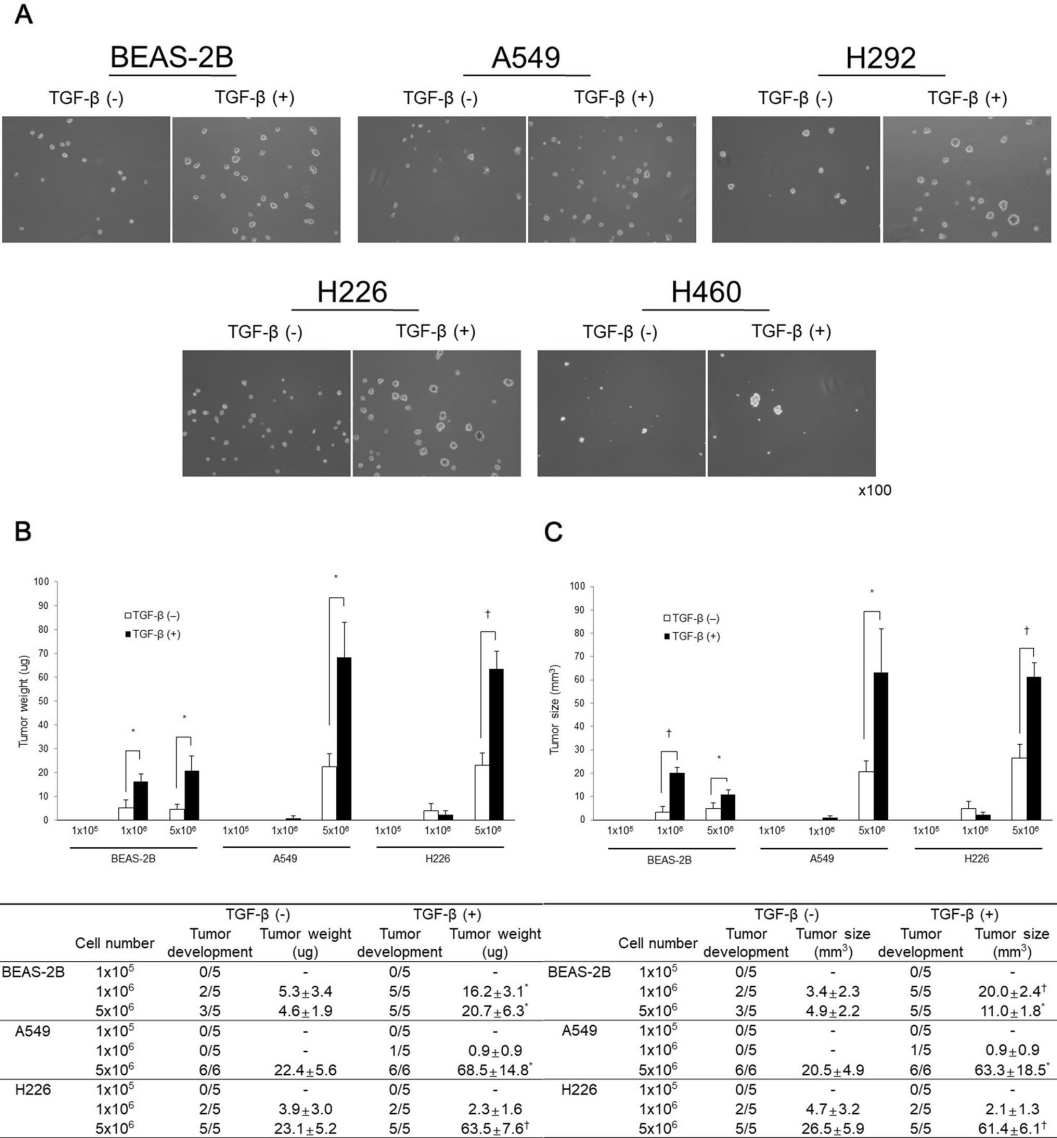
Acquisition of stemness in vitro and in vivo. (**A**) Sphere forming potentials of all lung cells following TGF-β treatment were higher than those of cells untreated with TGF-β. (**B**, **C**) Tumor formation abilities in BEAS-2B, A549, and H226 cells treated with or without TGF-β were compared following injection into BALB/c nude mice. Numbers of mice that developed tumors were higher in TGF-β treated cell groups compared to those in TGF-β untreated cell groups. Weight and size of the tumors were enhanced following injection of BEAS-2B (1 × 10^6^ and 5 × 10^6^), A549 (5 × 10^6^), and H226 (5 × 10^6^) cells treated with TGF-β compared with those in TGF-β untreated cell groups. Data are presented as mean ± SE (*P < 0.05, ^†^*P* < 0.01).

Sphere formation assay has been used to indicate stem cell growth. Sphere formation rates of all lung cells treated with TGF-β were increased compared to those untreated with TGF-β (Fig. 4A).

Tumor-forming ability of cells treated with TGF-β was compared following injection into BALB/c nude mice. The potential of lung cells to form a tumor was analyzed as the rate of tumor development based on size and weight. Tumor size and weight were higher in TGF-β treated cell groups than those in TGF-β untreated cell groups (Fig. 4B and C).

### Activation of slug and CD87 by its promoter demethylation

**Figure 5:**
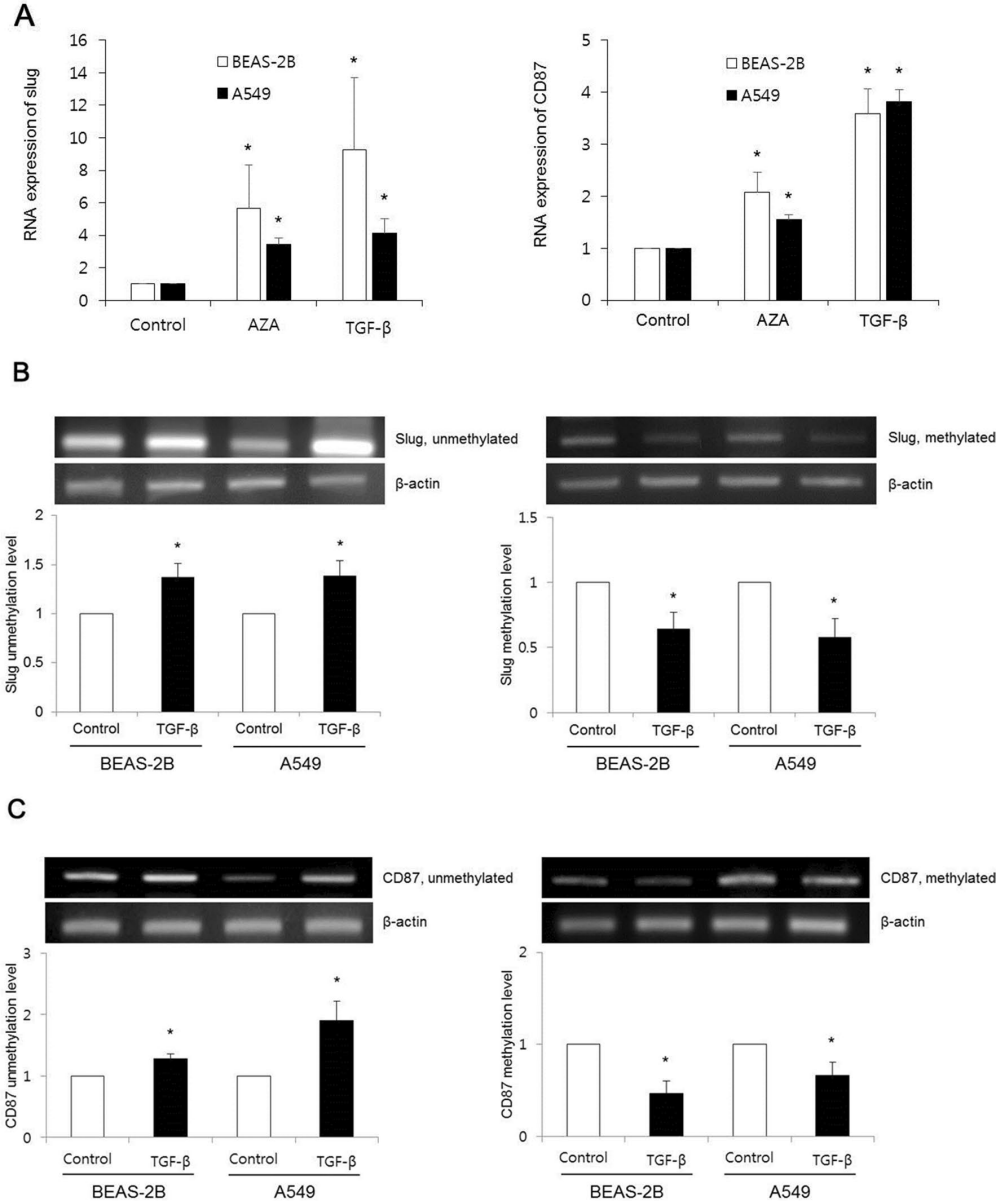
TGF-β induced slug and CD87 activations by their promoter DNA demethylations. (**A**) After treatment with either DNA methyltransferase inhibitor (AZA) or TGF-β, real-time RT-PCR indicated significantly enhanced slug and CD87 expressions compared with controls (non-AZA + non-TGF-β) in BEAS-2B and A549 cells. Data are presented as mean ± SE (n = 3, *P < 0.05). (**B**, **C**) Methylation-specific PCR revealed increased unmethylation levels of slug and CD87 genes in TGF-β treated cell groups whereas methylation levels of these genes were decreased following TGF-β treatment. These expression levels were compared with controls (non-TGF-β). Cropped images of Figs. B and C are displayed, uncropped blots are displayed in Supplementary Figs. S2 and S3. Data are presented as mean ± SE (n = 3, *P < 0.05).

To examine whether EMT and stem cell marker expressions were activated aberrantly, we investigated expression levels of EMT and stem cell markers after treatment with a DNA methyltransferase inhibitor AZA. AZA treated cells showed increased slug and CD87 expression levels compared with TGF-β (−) control (n = 3, *P* < 0.05; Fig. 5A). These findings suggest that DNA demethylation is associated with the activation of slug and CD87 expression.

DNA from lung cells was converted with sodium bisulfite. Unmethylation and methylation levels at slug and CD87 promoter regions were then compared among lung cells treated with or without TGF-β using methylation-specific PCR. After TGF-β treatment, unmethylation levels of slug and CD87 genes were increased whereas, methylation levels of slug and CD87 genes were decreased in both BEAS-2B and A549 cells (*P* < 0.05; Fig. 5B and C).

**Figure 6:**
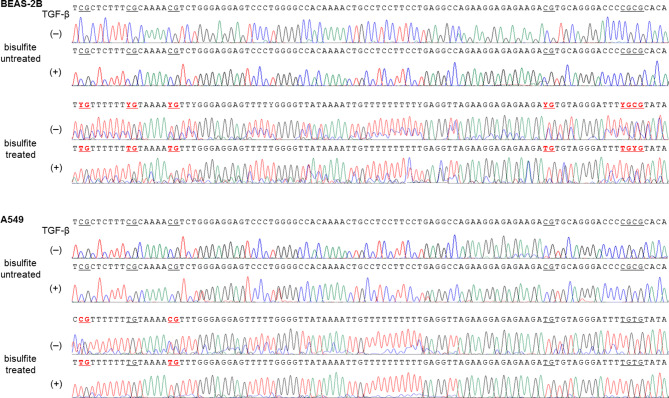
Representative demonstration of CpG site methylation changes of CD87 promoter region in BEAS-2B and A549 cells. Bisulfite sequencing showed six CpG sites presented in underlined letters within a 100-bp promoter region of CD87 gene. Demethylated CpG sites following TGF-β treatment are indicated by red letters. Heterozygote C/T double peaks are indicated by Y.

To indicate CpG site methylation pattern of CD87 promoter region, bisulfite sequencing was analyzed in BEAS-2B and A549 cells. Representative bisulfite sequencing results of six CpG sites (underlined letters) within a 100-bp promoter region of CD87 gene are presented in Fig. 6. After TGF-β treatment, demethylated CpG sites are indicated by red letters in Fig. 6. Heterozygote C/T double peaks (Y) were found at several sites.

## Discussion

The objective of this study was to examine the effect of TGF-β on possible EMT and stem cell markers in the induction of EMT and stemness acquisition. Moreover, we investigated whether the mechanism of EMT and stemness acquisition was related to the activation of possible EMT and stem cell markers by epigenetic change of related genes. Various lung cell lines were used because we want to confirm whether the effect of TGF-β in the EMT and stemness acquisition is a common feature despite different aggressiveness of lung cancer cell lines including normal immortalized lung cell line, BEAS-2B. We previously reported that CXCR4 activation by its aberrant promoter demethylation was associated with hypoxia-induced acquisition of EMT and cancer stem cell characteristics in lung cancer^16^.

In the present study, next-generation sequencing was performed for transcriptome analysis to investigate the effect of TGF-β on expression levels of EMT and stem cell markers. TGF-β induces the demethylation of H3K27me3 in Snail1 promoter and overexpresses Snail1, which cause EMT. Referring to these results, it’s possible that the mechanism of TGF-β to induce EMT is correlated with Smad, which is the upstream molecule of Snail in the TGF-β signaling cascade^21,22^. Based on previous evidences, the following candidate EMT and stem cell markers were chosen: EMT markers, E-cadherin, N-cadherin, fibronectin, vimentin, α-SMA, slug, snail, Twist1, Twist2, ZEB1, ZEB2 and ZO-1; stem cell markers, CD44, CXCR4, ABCG2, ALDH1A1, EpCAM, CD90, Nanog, SOX2, SSEA4, CD166, BMI-1, nestin, Musashi-1, CD87, MET, SLC3A2, c-Myc, and KLF4 (Table 1). Among these EMT markers, N-cadherin, fibronectin, vimentin, slug, and snail were enhanced. Results were validated by western blotting. Among these stem cell markers, CD44 and CD87 expression levels were increased. This result was verified by real-time RT-PCR. Experimental conditions including TGF-β concentration, duration of TGF-β exposure, and the type of cell lines might have influenced experimental outcomes.

TGF-β induced EMT was demonstrated by a reduction in epithelial marker (E-cadherin) and an induction in mesenchymal markers (N-cadherin, fibronectin, vimentin, slug and snail). These changes of EMT markers were functionally validated with wound healing and Matrigel invasion assay. All lung cell lines (i.e., normal cell line BEAS-2B and four cancer cell lines A549, H292, H226, and H460) displayed increased migration and transwell invasion. These results correspond with previous studies showing that TGF-β can induce EMT^23,24^.

Slug and snail are zinc finger transcription factors that regulate EMT in primary human cancers including pancreatic cancer, breast cancer, gastric cancer, lung cancer, and ovarian cancer^25–27^. Cell surface receptor CD44 and urokinase receptor CD87 can be candidate stem cell markers for multiple solid tumors including breast cancer, head and neck cancer, and ovarian cancer^28–31^. Although CD44 was analyzed to have 26 stemness signature, we could not find CD87 as a stemness marker from the StemChecker^32^. We designed our experiments to screen the stemness related genes with NGS, which was used by previous studies^30,33^ indicating CD87 might be a potential stemness marker in SCLC and breast cancer. The results of these studies provided the stemness genes and their physiological meaning, which are the main reason why we selected the stemness markers.

EMT marker slug can be induced by TGF-β treatment in prostate cancer and lung cancer^34,35^. Stem cell marker CD87 can be activated by TGF-β in melanoma cells^36^. Previous reports have suggested that expression levels of EMT markers (such as slug and snail) and stem cell markers (such as CD44) can be regulated by DNA methylation of promoter regions of these genes^13,37–39^. However, to the best of our knowledge, there have been few studies on DNA methylation associated with CD87 expression.

Methylation analysis was performed in cells exposed to TGF-β for 72 h to determine whether DNA methylation might play a role in EMT and stemness phenotype. DNA methyltransferase inhibitor AZA was used to evaluate whether TGF-β induced activation of EMT markers (slug and snail) and stem cell markers (CD44 and CD87) were related to epigenetic mechanism (DNA demethylation). We found that both TGF-β and AZA could activate expression of slug and CD87. However, AZA treatment did not activate expression of snail or CD44 (data not shown).

As far as we know, there have been few studies on the molecular mechanism of TGF-β induced EMT and stem cell markers or their functional relation in lung cancer. The present study confirmed that slug and CD87 were activated by TGF-β, which were related with their aberrant promoter demethylation.

The present study suggest that TGF-β-induced EMT and cancer stemness acquisition are related with slug and CD87 activation by their aberrant promoter demethylation. However, further study is required on the detailed epigenetic mechanism and the development of potential therapeutics for lung cancer.

## Supplementary information

Supplementary Information
