# Supplementary material for: TGF-β induced EMT and stemness characteristics are associated with epigenetic regulation in lung cancer
Source: Sci Rep. 2020 Jun 30;10:10597. doi: 10.1038/s41598-020-67325-7 (PMC7326979; doi:10.1038/s41598-020-67325-7)

TGF-β induced EMT and stemness characteristics are associated with epigenetic regulation in lung cancer

Bit Na Kim[1,2,#], Dong Hyuck Ahn[1,2,#], Nahyeon Kang[1,2], Chang Dong Yeo[1,2], Young Kyoon Kim[1], Kyo Young Lee[3], Tae-Jung Kim[3], Sug Hyung Lee[4], Mi Sun Park[5], Hyeon Woo Yim[5], Jong Y. Park[6], Chan Kwon Park[1,2,*] and Seung Joon Kim[1,2,*]

[1]Department of Internal Medicine, College of Medicine, The Catholic University of Korea, Seoul, Korea

[2]Postech-Catholic Biomedical Engineering Institute, College of Medicine, The Catholic University of Korea, Seoul, Korea

[3]Department of Hospital Pathology, College of Medicine, The Catholic University of Korea, Seoul, Korea

[4]Department of Pathology, College of Medicine, The Catholic University of Korea, Seoul, Korea

[5]Department of Biostatistics, Clinical Research Coordinating Center, The Catholic University of Korea, Seoul, Korea

[6]Department of Cancer Epidemiology, Moffitt Cancer Center, Tampa, FL, USA

[#]These authors contributed equally to this work.

[*]These authors also contributed equally to this work.

Address correspondence to Chan Kwon Park, MD, PhD

Department of Internal Medicine, St. Mary's Hospital, Postech-Catholic Biomedical Engineering Institute, College of Medicine, 222, Banpo-daero, Seocho-gu, Seoul, 06591, Korea

E-mail: ckpaul@catholic.ac.kr, Tel: 82-2-3779-1334

Address correspondence to Seung Joon Kim, MD, PhD

Department of Internal Medicine, Seoul St. Mary's Hospital, Postech-Catholic Biomedical Engineering Institute, College of Medicine, 222, Banpo-daero, Seocho-gu, Seoul, 06591, Korea

E-mail: cmcksj@catholic.ac.kr, Tel: 82-2-2258-6063

Supplementary figure 1. Uncropped blots from figure 2 of the manuscript

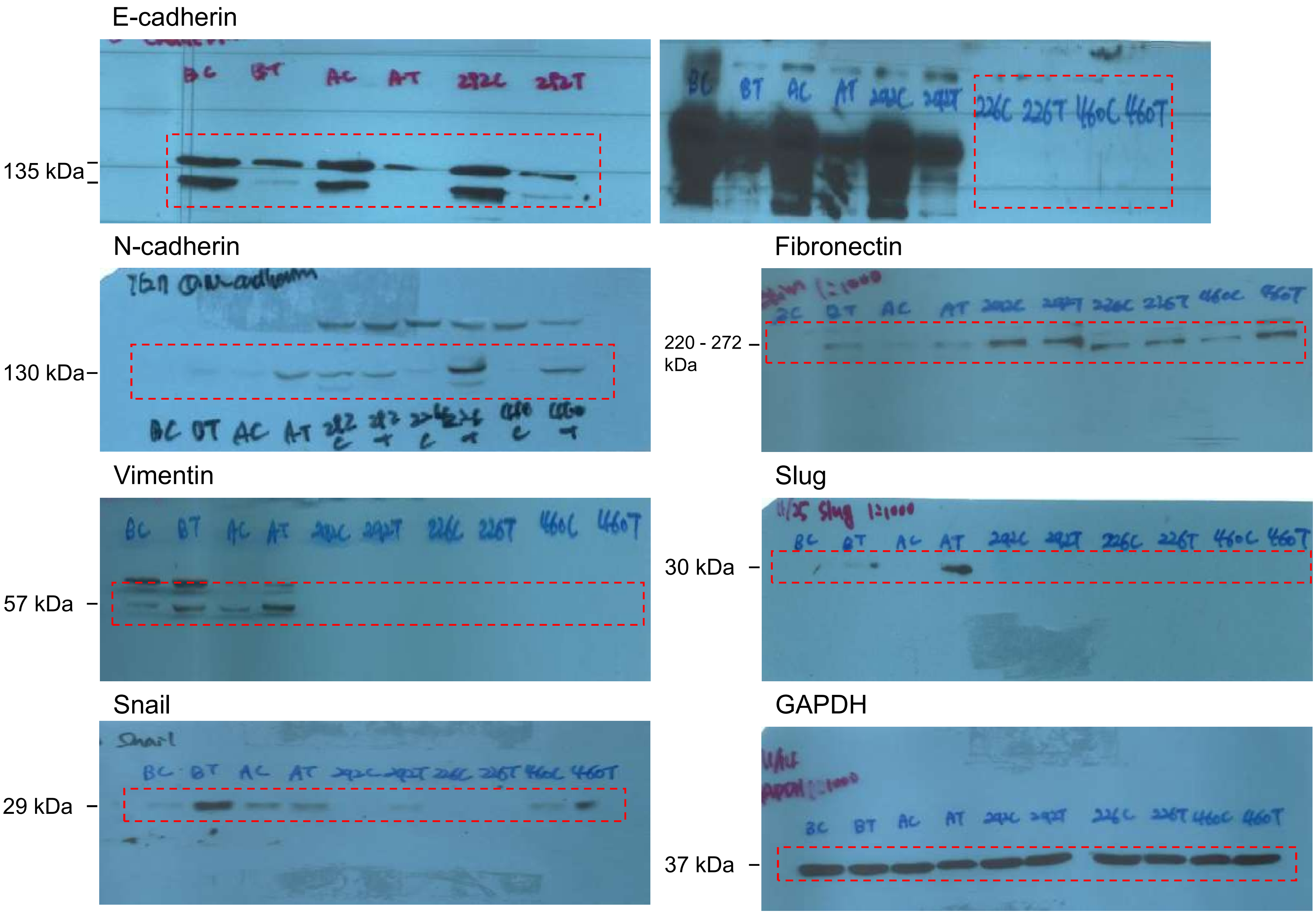

Supplementary figure 2. Uncropped blots from figure 5B of the manuscript

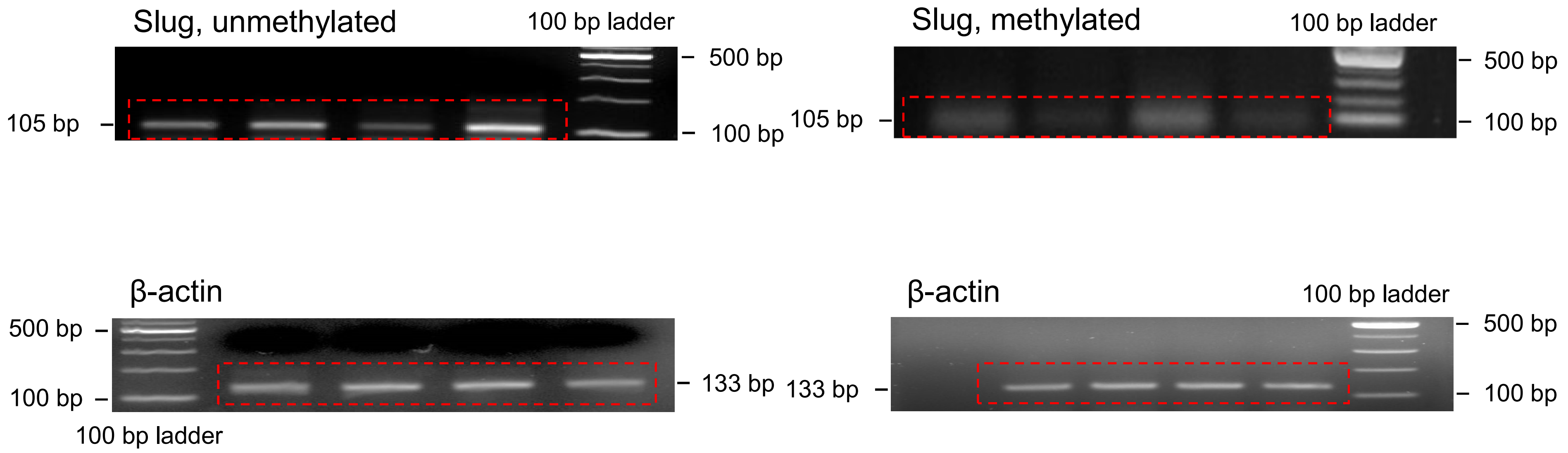

Supplementary figure 3. Uncropped blots from figure 5C of the manuscript

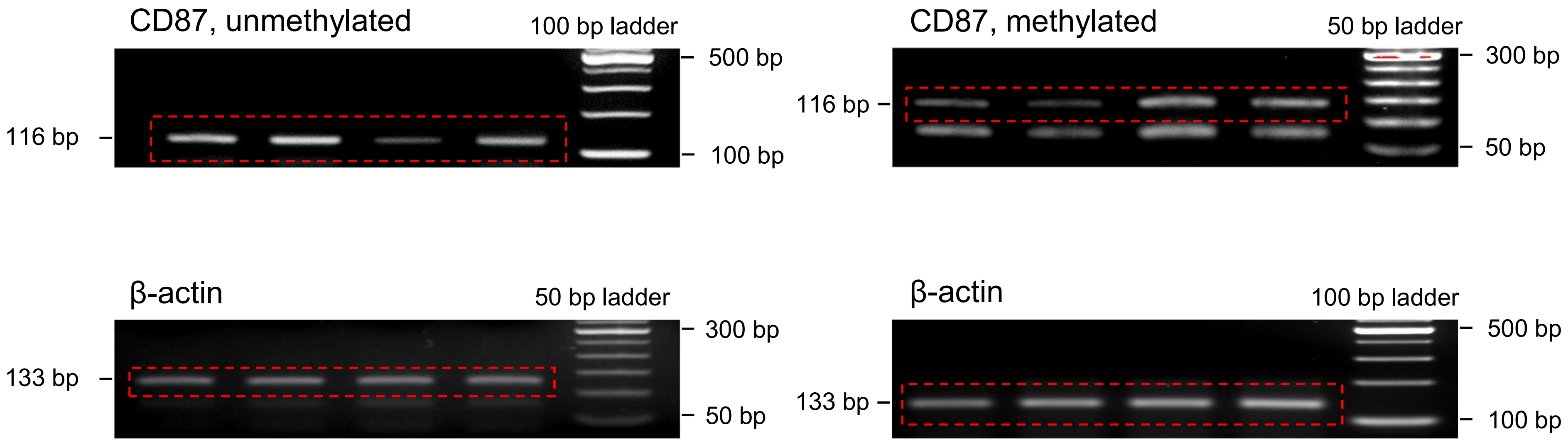

Supplement: Supplementary file 1 — Supplementary Information [file 41598_2020_67325_MOESM1_ESM.pdf]
